# Readiness for Use of HIV Preexposure Prophylaxis Among Men Who Have Sex With Men in Malawi: Qualitative Focus Group and Interview Study

**DOI:** 10.2196/26177

**Published:** 2021-10-27

**Authors:** Elizabeth Mpunga, Navindra Persaud, Christopher Akolo, Dorica Boyee, Gift Kamanga, Gift Trapence, David Chilongozi, Melchiade Ruberintwari, Louis Masankha Banda

**Affiliations:** 1 FHI360 Lilongwe Malawi; 2 FHI360 Durham, NC United States; 3 FHI360 Dar-es-Salaam United Republic of Tanzania; 4 FHI360 Monrovia Liberia; 5 Centre for Development of People Lilongwe Malawi

**Keywords:** MSM, readiness to use, PrEP, HIV prevention, qualitative assessment, HIV, men who have sex with men, Malawi, prophylaxis

## Abstract

**Background:**

Men who have sex with men (MSM) are a key group for HIV interventions in Malawi considering their high HIV prevalence (17.5% compared to 8.4% among men in the general population). The use of oral preexposure prophylaxis (PrEP) presents a new opportunity for MSM to be protected. We present the findings from a qualitative assessment designed to assess awareness of and willingness and barriers to using PrEP among MSM in Malawi.

**Objective:**

The 3 main objectives of this assessment were to determine: (1) awareness of PrEP, (2) factors that influence willingness to use PrEP, and (3) potential barriers to PrEP use and adherence among MSM in order to guide the design and implementation of a PrEP program in Malawi.

**Methods:**

Ahead of the introduction of PrEP in Malawi, a qualitative study using in-depth interviews (IDIs) and focus group discussions (FGDs) was conducted in October 2018 in Blantyre, Lilongwe, and rural districts of Mzimba North and Mangochi. With support of members of the population, study participants were purposively recruited from 4 MSM-friendly drop-in centers where MSM receive a range of health services to ensure representativeness across sites and age. Participants were asked what they had heard about PrEP, their willingness to use PrEP, their barriers to PrEP use, and their preferences for service delivery. The data were analyzed using a thematic content analysis framework that was predetermined in line with objectives.

**Results:**

A total of 109 MSM were interviewed—13 through IDIs and 96 through FGDs. Most participants were aware of PrEP as a new HIV intervention but had limited knowledge related to its use. However, the majority were willing to use it and were looking forward to having access to it. IDI participants indicated that they will be more willing to take PrEP if the dosing frequency were appropriate and MSM were involved in information giving and distribution of the drug. FGD participants emphasized that places of distribution and characteristics of the service provider are the key factors that will affect use. Knowing the benefits of PrEP emerged as a key theme in both the IDIs and FGDs. Participants highlighted barriers that would hinder them from taking PrEP such as side effects which were cited in IDIs and FGDs. Key factors from FGDs include cost, fear of being outed, drug stockouts, fear of being known as MSMs by wives, and lack of relevant information. FGDs cited stigma from health care workers, forgetfulness, and community associated factors.

**Conclusions:**

Despite having inadequate knowledge about PrEP, study participants were largely willing to use PrEP if available. Programs should include an effective information, education, and communication component around their preferences and provide PrEP in MSM-friendly sites.

## Introduction

Men who have sex with men (MSM) bear a disproportionate burden of the HIV epidemic [1]. In 2019, the Joint United Nations Programme on HIV and AIDS reported that gay men and other MSM accounted for 17% of all new HIV infections globally [2] and that their risk of HIV acquisition was 22 times higher than for heterosexual men. Similarly, in East and Southern Africa, MSM were reported to contribute to 17% of new HIV infections [2]. Data from studies conducted between 2011 and 2014 among MSM in Malawi found HIV prevalence ranging from 18.2% to 21.4% [3,4]. This is more than 3 times the national average of 6.4% among men ages 15 to 49 years in the general population [5].

In September 2015, the World Health Organization (WHO) recommended oral preexposure prophylaxis (PrEP) as part of a combination HIV prevention approach [6] for people at substantial risk of HIV infection. PrEP is the use of antiretroviral medication to prevent HIV-negative individuals from acquiring HIV. PrEP complements condom and lubricant use, behavioral counseling, treatment for sexually transmitted infections, HIV testing and counseling, voluntary male medical circumcision, and antiretroviral therapy (ART) for partners living with HIV [7-9]. A proportion of MSM do not use condoms consistently for various reasons; therefore, using PrEP during periods of higher risk of HIV responds to this reality and strengthens comprehensive HIV prevention [10]. Despite the evidence of its effectiveness in preventing HIV, access to PrEP has been limited in many countries. In Malawi, PrEP was included in the Malawi Guidelines for Clinical Management of HIV in Children and Adults 2016 and National HIV Prevention Strategy 2015-2020; however, at the time of this assessment, PrEP was not yet readily available in the country. This study was conducted ahead of the introduction of PrEP services in Malawi to inform national scale-up of PrEP services, especially for MSM. This assessment was conducted to support the design of the PrEP program in Malawi.

Numerous individual, familial, institutional, and structural factors affect MSM’s access to health care in Malawi. These include stigma from community members due to cultural beliefs [3]; hesitancy of MSM to reveal their sexual practices when accessing health care [4], which limits their ability to access appropriate services, information, education, and counseling; inadequate capacity of service providers to provide MSM-friendly health services [1-4]; and unfavorable social and legal environments created by the Malawi penal code that criminalizes same-sex relationships and encourages discrimination against MSM [11]. While MSM have a high turnover of sexual partners, low condom use, and multiple concurrent partnerships, they have reported low levels of individual risk perception [12,13]. However, it is important to note that these studies were conducted several years ago, and there is a need to conduct similar studies to obtain updated data on these risk factors. This discrepancy between actual and perceived risk requires programs to innovate and provide a wide choice of services to MSM to reduce the incidence of HIV among them [1].

Although the legal environment for MSM, transgender people, and other sexual minorities remains constrained in Malawi, significant progress has been made in the provision of health services. For instance, the current national (Ministry of Health) Clinical Guidelines for the Management of Sexually Transmitted Infections [14] and the Malawi National HIV Prevention Strategy 2015-2020 [15] have incorporated MSM-specific interventions. Civil society organizations, legal and advocacy partners, and national and international nongovernmental organizations, such as FHI360’s Linkages across the Continuum of HIV Services for Key Populations Affected by HIV (LINKAGES) project, are also providing a mix of human rights, legal, and HIV prevention services. LINKAGES was launched in June 2014 as the US President’s Emergency Plan for AIDS Relief (PEPFAR)/US Agency for International Development (USAID)’s largest global HIV project dedicated to providing holistic HIV prevention, care, and treatment programming for key populations. In Malawi, through community outreach services and drop-in centers (DICs), the project has been providing prevention, care, and treatment services to key populations, including MSM and transgender individuals.

The primary aim of this study was to assess MSM’s awareness of PrEP, their willingness to use it, and barriers they face to PrEP use in order to guide the design and implementation of a PrEP program in Malawi. Specifically, the study aimed to determine awareness and attitudes about PrEP, factors that will likely facilitate PrEP uptake and adherence, and potential barriers to PrEP uptake and adherence.

## Methods

### Study Design and Population

A qualitative study consisting of in-depth interviews (IDIs) and focus group discussions (FGDs) was conducted to ascertain MSM knowledge of and perceptions about PrEP uptake and adherence in Lilongwe and Blantyre cities and the two rural districts of Mangochi and Mzimba North. These districts were targeted as the project leveraged on the infrastructure of the LINKAGES project. Study participants were identified in October 2018 from MSM accessing HIV services from peer educators at various sites, including DICs supported by the LINKAGES project, as well through other projects run by the local civil society organization Center for Development of People (CEDEP). Prior to the study, MSM were engaged through CEDEP to provide input to the design and implementation of the study. Some of the input was on the questionnaire and language, who to include as data collectors, and where to conduct the IDIs and FGDs among others. Subsequently data collectors were picked from the group of MSM who participated in the consultation process. The LINKAGES-supported DICs are key population–friendly service delivery points where MSM access condoms, lubricants, HIV testing, and counseling; services for gender-based violence; and recreational activities. Men were eligible to participate in the study if they were aged 18 years or older, accessed services from LINKAGES-supported DICs or CEDEP projects, self-identified as MSM or were identified as such by peer educators, and tested negative for HIV within the past 3 months.

### Sampling

Study participants for FGDs were purposively recruited from men who accessed services at DICs to ensure representation from all sites and different age groups. Lists of eligible study participants were drawn both from LINKAGES DIC records and records of other CEDEP projects and were classified by geographical location, social networks, and age group. Participants were randomly selected within each subgroup. Clinical staff at the DICs were involved in the identification while ensuring that the participants met the eligibility criteria for the study.

A total of 8 FGDs with 12 participants per group were conducted making a total of 96 MSM participating in the FGDs. Two FGDs were conducted per district, including one for younger MSM ages 18 to 34 years and another for older MSM ages 35 years and older. A total of 13 IDIs were conducted, comprising 3 people from each of the 4 districts and one man who has sex with other men who was involved in advocacy. The IDI participants were purposively selected from LINKAGES projects and other social projects to include MSM who are highly hidden from society as well as the MSM community because of their social position. The 3 participants from each district had at least one of the following characteristics: married individuals, educated at a tertiary level or working, and older than 35 years.

### Data Collection

Data were collected in October 2018 following study approval. Data were collected by trained study assistants using semistructured interview guides. The study assistants were fluent in both English and Chichewa, a local language, and they conducted IDIs and facilitated FGDs in the language of each participant’s choice. Data collected from participants included information about their knowledge, perceptions, and concerns about PrEP; factors that would potentially motivate them to use it; barriers to PrEP use; and service delivery preferences related to infrastructure, knowledge needs, and providers. FGDs were done with 6 to 12 participants per group and conducted at DICs. Each FGD lasted for approximately 70 minutes, and 6 of the 8 FGDs were audiorecorded, as participants of 2 FGDs declined permission for the FGDs to be recorded. IDIs were conducted at a place convenient for the participant. On average, the interviews lasted 50 minutes, and 8 out of 13 interviews were audiorecorded, as 5 people declined to be audiorecorded. IDIs and FGDs used the same questions because the data collected in the IDIs supported the opinions collected in the FGDs from individuals that are highly hidden and could not participate in FGDs.

The 8 IDIs and 6 FGDs that were audiorecorded were transcribed into Chichewa or English by an independent transcriber, and all interviews conducted in Chichewa were then translated to English. Transcripts were proofread and checked for accuracy by listening to the recordings multiple times to ensure that the translation conveyed the participants’ answers and descriptions. The aim was to produce the best possible translation and preserve the content and meaning of the original text. For the 5 IDIs and 2 FGDs that were not audiorecorded, study research assistants took notes, including direct quotes.

### Data Analysis

Data were analyzed using a thematic content analysis framework, where deductive and inductive approaches were used to code the data. The former was guided by the literature and study objectives in identifying the themes under which data were grouped. Inductive analysis was then conducted by listening to the audiorecording, reading through the transcripts, and identifying emergent themes. The data analysis followed 6 stages [16]: becoming familiar with the data, generating initial codes, searching for themes, refining themes, naming themes, and producing the report [17]. The findings presented include quotes without identifiers to protect confidentiality.

### Ethics

The study protocol was reviewed by the University of Malawi, College of Medicine’s Research Ethics Committee and FHI360’s Protection of Human Subjects Committee. All study staff, including the study assistants, were trained in ethics for conducting research with human subjects, with a focus on ensuring informed consent, privacy, and confidentiality. They were also trained on data collection tools to standardize the information to be collected and the process of data collection. The data collectors were identified from the cohort of peer educators under the LINKAGES project who are part of the MSM community. Potential participants were informed that participation in the FGD and IDI was voluntary and that refusal will not affect continued access to services. Written informed consent was obtained from everyone who agreed to participate. Participants were not compensated for participating in the study but were reimbursed for the cost of travel from their home to the study site. No personal identifying information was collected during the FGD and IDI. Study participants were assigned IDs/unique identifiers, and no names were used. IDIs and FGDs were conducted in places that assured privacy where conversation between interviewer and interviewees and FGD facilitators and participants could not be heard by a third party.

## Results

### Sociodemographic Characteristics

Table 1 below summarizes the characteristics of the study participants. Most of the study participants were aged 35 to 40 years (30/109, 27.5%) and the least were aged 30 to 34 years (14/109, 12.8%). There was a uniform number of participants from the 4 participating districts.

**Table 1:** Characteristics of study participants (n=109).

Characteristics		Value, n (%)
**Age group (years)**		
	18-24	21 (19.3)
	25-29	17 (15.6)
	30-34	14 (12.8)
	35-40	30 (27.5)
	41+	27 (24.8)
**Education**		
	None	8 (7.3)
	Primary	37 (33.9)
	Secondary	50 (45.8)
	Tertiary	14 (12.8)
**Marital status**		
	Never married	44 (40.3)
	Married	44 (40.3)
	Separated	18 (16.5)
**District**		
	Lilongwe	28 (25.7)
	Blantyre	27 (24.8)
	Mangochi	27 (24.8)
	Mzuzu	27 (24.8)
**On salary**		
	Yes	30 (27.5)
	No	79 (82.5)

### Awareness and Attitudes Regarding PrEP

Of the 96 FGD participants, 40 indicated that they heard about PrEP before the study while 20 indicated that they heard about the drug during the survey. Of the IDI participants, 69% (9/13) had heard about PrEP prior the study and 4 learned about it during the study.

I hear it a drug you take before having sex to protect yourself from catching HIV.

FGD participant

It is a drug for HIV prevention which you take before you have sex with you partner.

IDI participant

When asked what they knew about PrEP, 35% (34/96) of the FGD participants and 67% (6/9) of the IDI participants said they did not know much. Of the FGD participants, 45% (18/40) who heard about PrEP confused PrEP with postexposure prophylaxis (PEP). Awareness was better among younger MSM participants than older MSM participants, as they were able to describe PrEP as prevention before acquiring HIV.

I have heard about PrEP of which I can’t describe well. I heard that these are drugs to be provided to HIV-negative people to protect them from HIV.

IDI participant

You use it to kill the virus within 72 hours of sleeping with someone without a condom.

FGD participant

When asked about the main source of their information on PrEP, the majority reported friends while others reported civil society organizations (CSOs) and social media. Family was not mentioned as one of the sources of information. There were some mixed ideas about the awareness and importance of using condoms while taking PrEP.

I heard about it this other day when I was drinking at a bottle store. I heard that when you do not have HIV you can take these drugs to protect yourself from HIV.

IDI participant

Participants from both IDIs and FGDs acknowledged the importance of using condoms when one is on PrEP to have double protection for HIV and prevent sexually transmitted infections. However, others pointed out that using condoms combined with PrEP defeats the purpose of using PrEP for HIV prevention as condoms similarly protect against HIV and other STIs.

Why should we use two things? I wish we could be choosing between the two.

FGD participant

Some participants were male sex workers and reported that condom use with clients was sometimes challenging due to client preferences. One participant described the appeal of PrEP in cases where he did not know clients’ HIV status.

I prefer having sex with a partner without using a condom. Sometimes we meet clients and we don’t have time to go for HIV test before sex. So if PrEP is available for use, I will be taking PrEP before having sex with someone whose HIV status I don’t know. PrEP will help me protect myself from HIV.

IDI participant

I wish the service could start immediately for us MSM like the female sex workers [FSWs] because PrEP will help me save my life as I don’t use condoms all the time because some clients prefer sex without a condom.

FGD participant

### Attitude

Despite the limited awareness of PrEP, there was overall a positive attitude toward PrEP. Study participants from both IDIs and FGDs noted that since PrEP will reduce their chances of being infected with HIV, the intervention was welcome within the MSM community. Of the FGD study participants, 67% (64/96) also indicated that they would not wait for a long time or for someone they knew to use PrEP before using it themselves, and they said they would use it right away. Some asked for the intervention to come to Malawi soon. From the IDIs, 38% (5/13) indicated that they would not wait to take PrEP but take it immediately as it is rolled out. A total of 62% (8/13) of FGD study participants indicated that they have multiple sexual partners and stated that they were more willing to use PrEP as an additional HIV prevention because sometimes they indulged in unprotected sexual intercourse with their partners.

Every time I am worried about getting infected with HIV. I want to have a long life. This initiative will make me healthy.

IDI participant

Let the drugs come, we are waiting.

FGD participant

Why would we wait for a year, were you trying us?

FGD participant

Once the drug is in we will use immediately, we can’t wait because the drug has already been tried in other countries.

IDI participants

### Benefits From PrEP

All 13 IDI participants indicated that if PrEP were available for them to use, they would want to use it to benefit from the protection from HIV. They mentioned preventing acquisition of HIV, enjoying a long life, and avoiding long-term ART if they otherwise get infected as benefits they would get from using PrEP. Another benefit was that PrEP would be able to prevent HIV transmission to sexual partners, including one’s wife. Likewise, 61% (52/96) of FGD participants both young and old indicated that HIV prevention will be their primary motivation.

I don’t know what I can do if I test HIV positive. It means I have to be on [antiretrovirals] daily. With PrEP maybe I would not be taking ART drugs daily.

IDI participant

If I protect myself from HIV I will not have to take ART for the rest of my life.

FGD participant

### Formulation and Dosing Preferences

Study participants were asked which of the different frequencies of dosing they were comfortable with and in what formulation (tablet or injection). Study participants were concerned about the dosing frequency and reported that they would be more likely to use PrEP if the dosage is not daily.

If we will be using daily tablets, it means we will have to go to the facility to collect medications so often. We do not have that kind of cash to be traveling all the time.

IDI participant

Weekly is good, because taking drugs brings bad taste in the mouth, so taking the drug daily for a long time is not good.

FGD participant

Preferences for oral or injectable PrEP were mixed. Some indicated that they would prefer an injectable form, while others wanted pills because they feared injections. However, some who were married indicated that they would like PrEP use to be disguised so that their spouses or parents would not suspect anything. Specifically, some participants expressed that they would prefer small pills, which would be less noticeable and easier to swallow.

I am married also to a woman. She may suspect something if I am using pills every day at home.

IDI participant

Once a month injection, because a daily pill is too difficult to be taken. And for it to be attractive, it must not have more side effects.

IDI participant

### MSM Involvement for PREP Demand Creation and Distribution

Participants also indicated the need to involve members of the MSM community to promote awareness of the product, as well as distribution of the drug. They pointed out the need to build the capacity of MSM representatives to share information about PrEP within the MSM community.

Choose MSM representatives from the MSM community to be like peer educators.

IDI and FGD participants

### FGD Participants Spelled Out Where It Is Provided and Who Provides It

Study participants were asked about their health services preferences that would contribute to PrEP uptake and adherence. DICs were the most preferred service delivery point, followed by outreach services. These settings were most often reported as venues where PrEP could be provided and accessed without concerns about stigma and discrimination. Many (30/96) participants from FGDs said they would prefer PrEP to be available in DICs and outreach sites, where there was minimal to no stigma and discrimination.

If you want MSM to take PrEP, then make sure that you provide services in places where we are already comfortable, like drop-in centers.

FGD participant

They also proposed changes such as improved attitudes for service providers, reduced waiting time, and provision of clear information about PrEP. Participants felt it was important for facilities to offer the following to accompany PrEP services: condoms and lubricants, HIV testing and counseling, and education on PrEP. Participants also said they would be more likely to adhere to PrEP if it was provided by nonjudgmental MSM-friendly service providers.

Use the friendly providers who do not take their personal beliefs to stop us from being MSM, those who understand that we are also human beings.

FGD participant

Two participants in IDIs reported a mission (church-affiliated) hospital in northern Malawi as especially well-placed to provide care for the MSM community, and private pharmacies were reported as potential sites by one person in an IDI.

### Potential Barriers for MSM to PrEP Uptake and Adherence

Despite the positive attitude and their willingness to take PrEP, the participants had several concerns that would stop them from taking PrEP which need to be considered during implementation of the services.

The potential side effects of PrEP were the most commonly mentioned concern in both IDIs and FGDs. When asked about the factors that will prevent them from accessing PrEP, 54% (7/13) of IDI participants and 23% (22/96) of participants from FGDs mentioned side effects.

They are medicines just like any other medicines, and inevitably there will be side effects. What are these side effects? … There could be smell or bad taste that one may have to deal with.

IDI participant

Medicines are medicines. Perhaps one may vomit after taking the medicines. That is why I think injections are better. I do not like the taste of medicines.

IDI participant

I have some fears. For example, what are the side effects of PrEP being a drug?

FGD participant

The costs (financial and time), fear of being identified as an MSM, insufficient information, dosage, and stockouts were identified in the IDIs as potential factors that can limit uptake of PrEP. Issues related to cost included transport costs to the point of care that could make it expensive to access PrEP through private community pharmacies. A total of 77% (10/13) of IDI participants indicated that it is important to use MSM peers as PrEP distributors, use male-only facilities, and target all men and not just MSM. Participants from IDIs also recommended the system should ensure that stock is consistently available.

The private pharmacies can be a good source of PrEP…. But nothing from these pharmacies is provided for free. We may not afford the cost of PrEP.

IDI participant

In public health facilities, PrEP should be provided to all men and not just MSM. For contraceptives, they give contraceptives to all women and not just female sex workers. In that case, there is no stigmatization.

IDI participant

If I go to facility and am sent back because drugs are out of stock, I will not go again because I am not sick.

IDI participant

You should package the drug in a way that people should not realize that it is a drug otherwise like for me I can’t take pills as it is difficult for me to keep them in the house because my wife can wonder and discover that I am an MSM.

IDI participant

The good thing that I see is best to take the pill once a month because it’s not easy to take drugs daily as people can be forgetful. ...and if you take once a month life will not be tough because when you take once a month you will take a long time before thinking of taking again which leaves you free.

IDI participant

If I don’t know how the drug works, its side effects and all other relevant information to help me make a decision, I wouldn’t bother to take it because it is drug I can’t be sure whether it will help me or harm me.

IDI participant

Common potential barriers that we identified among the FGDs included stigmatization of MSM by health care workers and community perception of persons on PrEP. Over 63% (60/96) of FGD participants reported experiencing stigma from health providers when they accessed sexual and reproductive health services, which could be similar when accessing PrEP services. They observed that when they want to access services in public health facilities, they are sometimes labeled by the providers as individuals who like sex “too much.” Three participants from the FGDs pointed out that it defeats the purpose of using PrEP if it cannot offer dual protection for STI and HIV like condoms.

If we go to get lubricants and condoms, the health providers think that you like sex too much.

FGD participant

Although some of the participants did not seem to care what the community would think, 22% (21/96) of FGDs participants reported concerns about how the drug is being advertised.

If the drug is for MSM and FSWs only and people know it, they will know that once you take it you are an MSM.

FGD participant

Why should we use two things? I wish we could be choosing between the two.

FGD participant

## Discussion

### Principal Findings

This study demonstrated that the majority of MSM in Malawi knew about the existence of PrEP and were enthusiastic about being able to access it. However, a significant number did not have many details about PrEP and only learned of it during the study. The overall level of understanding of PrEP seemed to be very limited. Formulation, frequency of dosing, place of distribution, and side effects were identified as key factors that can affect the uptake and continued use of PrEP. Participants of both the FGDs and IDIs indicated that MSM should be involved in the design of any education program promoting the use of PrEP.

A total of 42% (40/96) of persons from the FGDs and 69% (9/13) of participants of the IDIs indicated they had previously heard of PrEP. Common sources of information on PrEP included peers and the CSOs where they accessed services. Participants reported not having heard of PrEP from health care providers in public health facilities. This could be because PrEP was not yet available in public health services in Malawi. In addition, MSM normally access sexual and reproductive health services from MSM-friendly DICs owned by CSOs, whereas they attend public health facilities for services not related to sexual and reproductive health services. A total of 35% (34/96) of FGD participants and 46% (6/13) of IDI participants reported they had a limited knowledge of PrEP. Study participants were not aware that the WHO-approved PrEP regimen was oral dosing, and they questioned how or whether PrEP would work after unprotected sex with an HIV-infected partner. This finding suggests the need for adequate information for the MSM to understand the benefits of the drug and enhance uptake.

The MSM in this study demonstrated a positive attitude toward PrEP from both IDIs and FGDs despite the limited awareness and were enthusiastic about having another option for HIV prevention. A total of 67% (64/96) of FGD participants indicated that they would not wait to take the drug if it becomes available in Malawi. One FGD participant stated the following:

Let the drugs come, we are waiting.

FGD participant

The study identified factors that would enhance willingness for MSM to take PrEP. All participants from IDIs and 58% (56/96) of the FGD participants expressed that the benefits of PrEP will motivate them to take the drug. One IDI participant stated the following:

I don’t know what I can do if I test HIV positive. It means I have to be on [antiretrovirals] daily. With PrEP maybe I would not be taking ART drugs daily.

IDI participant

A quantitative study from the United States identified that not believing that it helps was one of the barriers that hinder MSM from being willing to take the drug [18]; therefore, this finding emphasizes the need for promoting awareness of the benefits of PrEP.

Other factors that promote willingness to take the drug include formulation and dosing of the medication with mixed expression with some liking the pills and others injection. In terms of dose, some did not care about daily dose while others preferred weekly dose. The responses could have been mixed because the participants were asked leading question between oral and injectable and among daily, weekly, and monthly doses, which could be different to reality where there are not many options. However, proper counseling will be needed to help MSM make the right choices and develop positive attitudes toward available formulation and dosage. MSM involvement in demand creation for the drug was another motivating factor as it will enhance peer motivation. Where the drug is provided and who provides it was another factor to enhance willingness. Considering that the medication is taken by people who are not sick, they may be easily put off if the environment of providing the service is not favorable. This is one of the key areas to address as many MSM face stigma and discrimination within the health care system and from the community [1-4]. Participants also expressed barriers to potential use of PrEP with the highest concern being about potential side effects expressed in both IDIs and FGDs. This may not appear to be a major issue for the program, since a review of the combination antiretroviral PrEP regimen TDF/emtricitabine taken as a single pill once daily showed that only 2 out of 15,678 participants discontinued therapy because of side effects [19]. However, it will be important to educate MSM about the potential side effects and how to manage them, and to address this perception regarding the side effects of the drug. Participants also discussed other barriers to potential use of PrEP: (1) lack of relevant information on PrEP, (2) the need to take PrEP daily, (3) potential stigma from public health facility providers, (4) stigma from the greater community if PrEP is thought to be used by MSM only, (5) forgetfulness, and (6) the costs (time and financial) required to access the drug. Study participants were also concerned that even if they themselves used PrEP, their partners may be exposed through their other partners, therefore there is need for universal partner education about PrEP. However, these concerns are not unique to MSM in Malawi, as these similar issues were noted in a review of 18 randomized controlled trials on PrEP: stigma, an unacceptable dosing regimen, side effects, low risk perception, low decision-making power, and the logistics of daily life [20]. In another PrEP-related study from Kenya, there were concerns around the competency of service providers to understand the multifaceted issues that key populations face [21]. This relates to the finding in our study that it is important to understand the needs of MSM before implementing PrEP services to promote uptake of services. These concerns may result in nondaily use of PrEP [22], thereby providing less-than-optimal protection. PrEP programs will need to develop and implement strong information and education interventions to ensure that high levels of knowledge among PrEP users are achieved.

The new PrEP program in Malawi needs to use a multifaceted approach to educating MSM about PrEP and ensuring access to PrEP. This may include dissemination of information through MSM-friendly service providers who understand their needs, use of MSM peers as educators and distributors of PrEP, and use of DICs to deliver services as well as male-only facilities such as barber shops for information sharing—when using public facilities they should target all men and not just MSM. There will need to be various modes of delivering the messages such as face to face interaction, printed materials like leaflets, and online platforms among others.

While we expected that MSM would mention the MSM-friendly CSO where some of the participants were recruited as a preferred setting to receive PrEP without stigma and discrimination, outreach settings were also reported as alternatives, especially for the highly hidden populations who do not go to DICs. Nevertheless, distance to the dedicated sites was identified as a bottleneck, suggesting that a combination of DICs and outreach facilities may be ideal. Hidden participants who do not go to DICs were most likely to mention public and private static health services, including community pharmacies, as settings where PrEP could be offered by MSM-friendly staff. However, it is important to note that a mission hospital in one of the districts was mentioned as a preferred site. This observation is somewhat counterintuitive because of the common narrative that religious facilities exhibit homophobic tendencies. This serves as a reminder that while it is possible to broadly categorize potential service providers as supportive or not supportive, individual preferences may also come into play.

Our study pointed out the need to minimize stigma and its effects in the environment where PrEP will be provided as a way of ensuring accessibility. The stigma is usually from other members of the community and MSM themselves. PrEP services will therefore need to be offered in settings that are stigma-free to accommodate this initiative. Community readiness is often defined as the various attributes of a community’s context that constitute a prerequisite to the implementation of effective change. Thus, the affected community’s knowledge and attitudes and its capacity to implement change strategies before an intervention is implemented should be reviewed [22,23]. It is important to take into consideration the readiness of community members other than MSM to accommodate the provision of PrEP services to avoid the fear of stigma underscored in this assessment.

### Limitations

Since the study was qualitative with nonprobability sampling, it was not designed to quantify the proportion of MSM in the study area who find PrEP acceptable. The findings may not be generalizable beyond the study setting and study groups because the study largely included MSM who accessed health services through the LINKAGES project or other projects within the same coverage and did not include MSM who are not obvious and regular health and social service users. However, IDIs included MSM who are more hidden and access health and other social services through other projects within CEDEP. Data were collected through self-report and so may be influenced by social desirability bias (where study participants respond to questions in a manner they believe the researchers want to hear). However, the risk of this bias was mitigated by the research assistants not being health professionals and by the fact that they, too, were members of the MSM community; therefore, there was less likely to be a power differential between the study participants and the study assistants. While a few key quotes may have been missed from the 2 FGDs and 5 IDIs that were not recorded, we do not believe that this significantly affected the overall conclusion from the study.

### Conclusion

The study provided insights on areas to pay attention to when designing a PrEP program for MSM in resource-constrained settings where MSM are criminalized like Malawi. It is critical to engage the MSM community to develop MSM-appropriate and relevant educational and informational materials on PrEP to address most of the concerns raised in this study and possibly promote both uptake and adherence. PrEP counseling should include, but not be limited to, information on side effects and how to manage them and the expected duration of side effects. Awareness-raising (information, education, and communication) activities should involve MSM as champions and provide clear information on the importance and value of taking PrEP in relation to the perceived risk of contracting HIV to promote adherence, regardless of PrEP formulation. Making community-based distribution of PrEP more prominent could avoid potential accessibility barriers, but it is critical to also consider how to target PrEP within the general population to reduce stigma and the perception that PrEP is only for MSM and other key populations. Furthermore, implementers should consider development of innovative public-private partnerships and explore how MSM-friendly health workers in the public and private health system may be mobilized and trained for the provision of PrEP in addition to usual safe spaces such as DICs. Other suggested factors necessary to improve access through health facilities should also be addressed (eg, drug stock, waiting time, and key population–friendly services).

## Abbreviations

ART: antiretroviral therapy
CEDEP: Center for Development of People
CSO: civil society organization
DIC: drop-in centers
FGD: focus group discussion
FSW: female sex worker
IDI: in-depth interview
LINKAGES: Linkages across the Continuum of HIV Services for Key Populations Affected by HIV
MSM: men who have sex with men
PEP: postexposure prophylaxis
PEPFAR: US President's Emergency Plan for AIDS Relief
PrEP: preexposure prophylaxis
USAID: US Agency for International Development
WHO: World Health Organization
